# Spatial distribution and determinants of barriers of health care access among female youths in Ethiopia, a mixed effect and spatial analysis

**DOI:** 10.1038/s41598-023-48473-y

**Published:** 2023-12-06

**Authors:** Elsa Awoke Fentie, Desale Bihonegn Asmamaw, Wubshet Debebe Negash, Tadele Biresaw Belachew, Tsegaw Amare Baykeda, Banchlay Addis, Tadesse Tarik Tamir, Sisay Maru Wubante, Abel Endawkie, Alebachew Ferede Zegeye, Atitegeb Abera Kidie, Samrawit Mihret Fetene

**Affiliations:** 1 Department of Reproductive Health, Institute of Public Health, College of Medicine and Health Sciences, University of Gondar, Gondar, Ethiopia; 2 Department of Health Systems and Policy, Institute of Public Health, College of Medicine and Health Sciences, University of Gondar, Gondar, Ethiopia; 3 School of Public Health, The University of Queensland, Brisbane, Australia; 4 Department of Pediatric and Child Health, School of Nursing, College of Medicine and Health Sciences, University of Gondar, Gondar, Ethiopia; 5 Department of HI Health Informatics, Institute of Public Health, College of Medicine and Health Sciences, University of Gondar, Gondar, Ethiopia; 6 Department of Epidemiology and Biostatistics, School of Public Health, College of Medicine and Health Science, Wollo University, Dessie, Wollo Ethiopia; 7 Department of Medical Nursing, School of Nursing, College of Medicine and Health Sciences, University of Gondar, Gondar, Ethiopia; 8 School of Public Health, College of Health Science, Woldia University, Woldia, Ethiopia

**Keywords:** Health care, Medical research

## Abstract

Access to healthcare services is a fundamental human right for every citizen, and it is the responsibility of the nation to guarantee that these services are acceptable, easily accessible, and timely. Barriers to accessing health services may have a detrimental effect on an individual’s physical, and mental health, and overall quality of life. However, access to health care services is a common problem in developing countries. Therefore, this study aimed to investigate spatial distribution and determinants of barriers to healthcare access among female youths in Ethiopia. Secondary data analysis was conducted based on the Demographic and Health Surveys data conducted in Ethiopia. A total weighted sample of 6143 female youths aged 15–24 years old was included in this study. A mixed-effect analysis was employed to identify factors contributing to barriers to healthcare access among youths in Ethiopia. Adjusted Odds Ratio with 95% CI was used to declare the strength and significance of the association. The concentration index was used to assess wealth-related inequalities, while spatial analysis was used to explore the spatial distribution and significant windows of barriers to healthcare access. This study revealed that the magnitude of barriers to healthcare access among female youth was 61.3% with 95%CI (60.1 to 62.5) to at least one or more of the four reasons. Age 15–19 years old (AOR = 0.80, 95%CI 0.68 to 0.95), no formal education (AOR = 2.26, CI 1.61, 3.18), primary education (AOR = 2.21, CI 1.66, 2.95), marital status (AOR = 1.43, 95% CI 1.21, 1.70), poor household wealth (AOR = 1.63, 95% CI 1.31, 2.05), no Media exposure (AOR = 1.67, 95%CI 1.41–1.98), reside in rural areas (AOR = 1.63, 95%CI 1.05 to 2.54), and low community media exposure (AOR = 1.45, 95%CI 1.01–2.08) were significantly associated with barriers of health care service. Barriers to healthcare access were significantly and disproportionately concentrated in poor households. A non-random Barrier to healthcare access was observed in Ethiopia. Among the 9 regions, primary clusters were identified in only 4 regions (North Ormiya, Benishangul Gumuz, Gambella, and South Nation Nationality and Peoples regions. A significant proportion of female youths faced barriers to health care access Age, educational status, marital status, rural residency, low economic status, and media exposure were factors associated with barriers to health care access. Therefore, program planners and decision-makers should work on improving the country’s economy to a higher economic level to improve the wealth status of the population, promote media exposure, and increase access to education.

## Introduction

The World Health Organization (WHO) defines youth as an individual in the age group 15–24 years^1^. It is the transitional stage from childhood to adulthood with biological, social, and psychological change, so it is a time of risk and opportunity for their future life^2^. Health is vital for having a socially and economically productive life^3^. Access to healthcare has an impact on an individual's overall health, including their physical, emotional, and social well-being. To achieve and maintain good health, prevent and treat diseases, reduce the risk of disability and premature death, and achieve health equity, one must have access to comprehensive, high-quality treatment^4^. According to the World Health Organization (WHO), access to healthcare services is a fundamental human right for every citizen, and it is the responsibility of the nation to guarantee that these services are acceptable, easily accessible, and timely^5^.

Within a total global population of 1.86 billion people aged 10–24 years, there were around 1·49 million deaths in 2019. Just under half of these deaths occurred in people aged 20–24 years and—one-third occurred in people aged 15–19 years. Most adolescent deaths in both sexes occurred in South Asia and sub-Saharan Africa. HIV/AIDS and sexually transmitted infections were the leading causes of death for female adolescent age groups in sub-Saharan Africa. Maternal death was still the—third most common cause of death for females aged 20–24 years in Africa, the Middle East, and South Asia, and it was also the fourth most common cause of death in Latin America and the Caribbean^6^.

Barriers to accessing health services may have a detrimental effect on an individual’s physical, and mental health, and overall quality of life^7^. Regrettably, access to health care services is a common problem in developing countries^8^. Around eight million deaths worldwide are caused by treatable medical problems, and 400 million people lack access to healthcare services^9^. Only half of the population in Africa had access to modern healthcare facilities^10^, primarily because of financial constraints and distance from such facilities^11^.

Evidence shows that socioeconomic factors, such as women and their husbands’ educational level, household economic status, place of residence, language barriers, occupational status, autonomy of women, and parity affect women’s access to health services^12–14^. Women face greater difficulties in getting the health care they need. Furthermore, gender-based limit the ability of women to protect their health and achieve optimal health status^15^.

Sustainable Development Goals (SDG) were introduced in late 2015 to ensure healthy lives and well-being for all people of all ages^16^. Related to this, The majority of sub-Saharan African countries have universal health coverage as one of their national health policies.

However, there is still little evidence that this commitment is being transformed into observable results through the mobilization of the national budget in the areas of health, and financial protection^17^.

Additionally, the Ethiopian government has prioritized youth and adolescent health initiatives over the past 20 years, particularly those that address sexual and reproductive health (SRH) and youth development^18^. However, youths in Ethiopia continue to have a high burden of morbidity and mortality due to a variety of factors, including adolescent pregnancy, unplanned pregnancy, poor nutrition, sexually transmitted infections (STIs), unsafe abortion, early marriage, and unmet needs for family planning^19^.

Even though there are studies about barriers to health care access, there is no evidence at the national level regarding the distribution of the problem in different geographic areas and the contributing factors to barriers to healthcare access among female youths in Ethiopia. It is essential for providing updated information and designing geographically focused interventions. It is also important to understand what factors influence improving healthcare access and reducing morbidity and mortality caused by the lack of healthcare access. Therefore, this study aimed to answer the following research questions. What was the magnitude of barriers of Health care access among female Youth in Ethiopia in 2016 EDHS? Are there wealth-related inequalities of barriers to healthcare access? What are the individual and community-level factors that contribute to barriers to healthcare access? What is the spatial distribution of barriers to health care access?. The findings of this research will be valuable for policymakers and program planners as preliminary evidence to plan and decide accordingly. Evidence is also very important to identify high-risk areas, implement targeted interventions, and evaluate the effectiveness of control measures.

## Method

### Study design, setting, and data source

This study was based on secondary data analysis from the 2016 Ethiopia Demographic and Health Survey (EDHS). The surveys were carried out cross-sectionally within 5-year intervals at the national level. The primary goal of the Ethiopia Demographic and Health Survey (EDHS) is to produce new health and health-related indicators. Ethiopia is a nation in East Africa that is located on the continent's horn of Africa. The country is administratively subdivided into nine regional states such as Afar, Amhara, South Nation Nationality and People’s (SNNP) Region, Benishangul-Gumuz, Gambela, Harari, Oromia, Somalia, Tigray, and two city administrations (Addis Ababa and Dire Dawa)^20^.

### Population and sampling technique

The study participants were selected using a two-stage stratified cluster sampling procedure. Stratification of regions into urban and rural areas was considered. In the first stage, 645 Enumeration areas (EAs) (202 from urban areas) were selected using probability sampling proportional to the EAs size and with independent selection in each sampling stratum. In the second stage, 28 households from each cluster were selected with an equal probability of selection from the household listing. The study participants from each Enumeration area (EA) stratum were selected independently by using the probability sampling technique^21^. The detail of the methodology is available in the full report of the 2016 EDHS. A total of 6143 weighted female youths^15–24^ were included in the study.

### Study variable

The outcome variable in this study is Barriers to accessing health care. It is a composite variable based on four questions related to challenges in accessing healthcare, such as difficulties in obtaining money, distance to health facilities, permission to consult with a doctor, and concerns about going alone. Women who encounter at least one of these challenges are classified as having barriers to healthcare access. In contrast, those who do not face any of these challenges are considered to have no barriers to accessing healthcare^22^.

The independent variables of this study comprised individual-level variables including age, sex, marital status, educational status, family size, wealth index, media exposure, and health insurance, and community-level variables including place of residence, region, community media exposure, and community poverty. The socioeconomic status was measured using the wealth index from Demographic Health Survey (DHS) data sets. In the DHS data, the wealth index was constructed using principal component analysis and then categorized as poorest (quintile 1), poorer (quintile 2), middle (quintile 3), richer (quintile 4), richest (quintile 5)^23^. media exposure (media exposure was created from the three variables: watching television, listening to the radio, and reading a newspaper, and labeled as yes if a woman has exposure to either of the three media sources or no if a woman has exposure to none of them^6^.

Based on the development status and the need for governmental support, the 11 regions of Ethiopia are categorized into three groups; ‘three Metropolis’ (Addis Ababa, Harari, and Diredewa), large central (Tigray, Amhara, Oromia, SNNPR), and “small peripherals” (Afar, Benshangul-Gumuz, Gambela, and Somali^24^. The community poverty level was the proportion of women in the poorest and poorest quintiles at the cluster level. Community-level media exposure was the proportion of women who had been exposed to at least one media (television, radio, or newspaper). The aggregated community-level variables were categorized as low and high based on a national median value^25^.

### Data management and analysis

The EDHS data used in this study was obtained from http://www.measuredhs.com, the official DHS measure website. The outcome and the independent variables were extracted from the set of IR data. Stata 14 was used to recode the data and conduct the analysis. Weighting was done to restore the sample's representativeness; as a result, the overall sample now closely resembles the nation's actual population.

Categorical variables were reported using frequency and percentage; continuous normal variables were reported using mean; and continuous explanatory factors that deviate from normality were reported using a median followed by an interquartile range.

### Multi-level analyses

We predict that female youths in the same cluster will be more similar to female youths nationwide because the EDHS data have a hierarchical structure with individuals nested within clusters. This means a multi-level model should be used to account for the variability between clusters. Therefore, to assess the clustering effect of barriers to healthcare access, mixed-effect model was fitted with a cluster-level random intercept. The random effect parameters were considered to measure the variation of barriers to accessing health care across communities or clusters, which were assessed using intraclass correlation coefficient (ICC), median odds ratio (MOR), and a proportional change in variance (PCV). Finally, model fitness was checked using deviance.

The mixed effects were used to estimate the association between the likelihood of barriers to accessing health care and explanatory variables at both individual and community levels. From bivariate analysis factors with a p-value of 0.2 were selected as candidates for the final model. In the final multivariate analysis, the associations between dependent and independent variables were presented using adjusted odds ratios (AOR) and 95% confidence intervals (CI) with a p-value of < 0.05. Generally, four models were fitted. These are the null model (a model containing only the outcome variable), model 1 (a model with the outcome variable and individual-level variables), model 2 (a model with the outcome variable and the community variables only), and model 3 (a model with the outcome variable and both the individual and community level variables Model fitness as assessed using information criteria. The best-fitted model was selected as a model with a larger log-likelihood ratio test, lowest AIC, and BIC.

### Concentration index and graph analysis

A concentration curve was used to determine whether socioeconomic inequality in some health variable exists or more pronounced at one point than another^26^ and the concentration index was used to quantify and compare the degree of socio-economic related inequality in a health variable^27,28^. The concentration index is twice the area between the concentration curve and the line of equity with the range of − 1 to + 1 and the sign indicates the direction of the relationship between barriers to healthcare access and the distribution of living standards (wealth status). Accordingly, concentration index = 0 indicated the distribution was proportionate, concentration index = 1 displayed that the richest person had all of the health variables, whereas concentration index = − 1 indicated that the poorest person had all of the health variables)^29,30^. The present study used Erreygers normalized concentration index (ECI) which is a modified version of the concentration index^31^.

### Spatial analysis

The weighted frequency of the outcome variable with cluster number and geographic coordinate data was used. A total of 611 clusters having longitude and latitude were included in the spatial analysis. Spatial autocorrelation analysis (Global Moran’s I index), spatial distribution, incremental autocorrelation, spatial interpolation, and detection of hot spot areas were conducted by using ArcGIS 10.7, while spatial scan statistics were done by SaTscan 9.6.

#### Spatial autocorrelation analysis

The Global Moran’s I statistic test was used to measure whether barriers to healthcare access were randomly distributed, dispersed, or clustered in Ethiopia. The calculated Moran’s I value close to − 1 indicates barriers to healthcare access were dispersed, whereas Global Moran’s I close to + 1 indicates barriers to healthcare access were clustered, and if the Global Moran’s I value zero barriers to healthcare access were distributed randomly^32^.

*The spatial interpolation* technique was used to predict barriers to healthcare access in unsampled households based on sampled clusters. The geostatistical ordinary kriging spatial interpolation technique was used for the prediction of unsampled clusters using ArcGIS 10.7 software^25^. Different interpolation methods are available. One method is Kriging, which is a geostatistical interpolation method that considers both distance and degree of variation between known data points when estimating values in unknown areas. In this study, ordinary Kriging was used as compared to Universal Kriging. The ordinary one is the basic form and shows the spatial correlation between the data and also determines the weights. Universal kriging describes the trend change of the predicted variable in a certain spatial neighborhood by establishing the functional relationship between the predicted variable and explanatory variables.

#### Hot spot analysis

Getis Ordi Gi's statistical hotspot analysis was done to identify the significant high and low cluster areas. This study identifies hot spot areas (areas with high rates of barriers to health care access) and low cold spot areas (areas with lower rates of barriers to health care access). Interpretations were made based on the z-score and p-value. A significant positive z-score, the larger the z-score is a clustering of high values (hot spot areas or high prevalence of barriers to health care access), and a significant negative z-score, the smaller the z-score indicates a clustering of low values (cold spot areas or low prevalent areas).

#### A spatial scan statistical analysis

We conducted a Bernoulli-based spatial scan statistical analysis to detect the geographical locations of statistically significant clusters for barriers to healthcare access households using Kuldorff’s SaTScan version 9.6 software^33^. The spatial scan statistics use a circular scanning window that covers statistically significant spatial clusters in barriers to health care access. To fit the model female youths who had barriers to healthcare access were taken as cases and those who had no barriers were taken as controls. The circle with the highest LLR test statistic was defined as the most likely (primary) cluster. For each identified cluster, the log-likelihood ratio (LLR) test statistic with its p-value, the relative risk (RR), the location radius, population, and cases were reported.

### Ethical consideration

This study is data from the DHS program, so it does not require ethical approval. However, online registration and requests for measure DHS were conducted to access the data. The dataset was downloaded from the DHS online archive (http://www.dhsprogram.com) after getting permission. All methods were carried out following the Declaration of Helsinki.

## Result

### Socio-demographic characteristics

A total of 6143 youths were included in the final analysis of the study. The median age of youths was 19 with (IQR: of 17 to 21) years. More than three-four (76.1%) youths were rural dwellers, 54.25% had primary education, 56.97% were not married, and 32.98% poorer wealth index (Table 1).

**Table 1:** Socio-demographic characteristics of female youths in Ethiopia, 2016 (n = 6143).

Variables	Categories	Weighted frequency	Percentage (%)
Age	15–19	3381	55.04
	20–24	2761	44.96
Residency	Urban	1467	23.89
	Rural	4676	76.11
Educational level	No education	1230	20.03
	Primary	3333	54.25
	Secondary	1184	19.28
	Higher	396	6.44
Occupation	Not working	3628	59.06
	Working	2515	40.94
Marital status	Married	2643	43.03
	Unmarried	3500	56.97
Wealth index	Poorest	945	15.39
	Poorer	1081	32.98
	Middle	1114	18.13
	Richer	1229	20.01
	Richest	1774	28.88
Media exposure	No	3039	49.47
	Yes	3104	50.53
Covered by health insurance	No	5843	95.11
	Yes	300	4.89
Region	Metropolitan	456	7.42
	Large central	5360	87.26
	Small peripheral	327	5.32
Community level poverty	Low	3346	54.47
	High	2797	45.53
Community level media exposure	Low	3626	59.03
	High	2517	40.97

### The magnitude of barriers to healthcare access

In this study, more than two-thirds (61.3, 95%CI 60.1to 62.5) of youths had at least one barrier to accessing health care, of which getting money (46.27%) and distance of health facility (42.10%) were the most frequently mentioned challenges.

#### Random effect analysis and model comparison

The ICC value in the null model indicates that 36.6% of the total variations in barriers to healthcare access were due to the difference between clusters. Besides, the high MOR value in the null model which was 3.97 revealed that when we randomly selected youths from two clusters, youths from a high-risk cluster had 3.97 times more likely to have barriers to healthcare access as compared to youths from a low-risk cluster. Moreover, the PCV in the final model revealed that about 32.8% of the variability in barriers to healthcare access was explained both by individual and community-level factors. Regarding model fitness, model 3 was the best-fit model since it had the lowest deviance or highest log-likelihood(Table 2).

**Table 2:** Random effect model and model fitness for the assessment of barriers to healthcare access among females in Ethiopia in 2016.

Parameter	Null model	Model 1	Model 2	Model 3
ICC	36.6	29.8	28.4	27.98
MOR	3.97 (3.27, 4.26)	2.97 (2.66, 3.38)	2.94 (2.63, 3.38)	2.92 (2.62, 3.31)
PCV		30.5%	32.1%	32.8%
Model comparison				
Log-likelihood	− 3297.9762	− 3224.4759	− 3166.7364	− 3145.8247
Deviance (-2LL)	6595.9524	6448.9518	6333.4728	6291.6494
AIC	6599.952	6463.88	6357.975	6324.425
BIC	6613.481	6511.229	6439.146	6423.557

### Mixed effect analysis of factors associated with barriers to health care access

All variables that had a p-value < 0.20 in the bivariate analysis were eligible for multivariate analysis. Based on the final model result, individual-level variables such as age, educational status, marital status, wealth index, media exposure, residence, and community-level media exposure were significant predictors.

Female youths aged 15–19 years were 20% less likely to have barriers to healthcare access than aged 20–24 years (AOR = 0.80, 95%CI 0.68 to 0.95). Compared with youths with a higher educational status, those with no formal education and primary education had 2.26 (AOR = 2.26, CI 1.61, 3.18) and 2.21(AOR = 2.21, CI 1.66, 2.95) times higher odds of barriers of healthcare access respectively. The likelihood of having barriers to accessing health care among unmarried youths was 1.43 (AOR = 1.43, 95% CI 1.21, 1.70) times higher compared with married youths**.** Similarly, the odds of barriers to healthcare access among poor youths were 1.63(AOR = 1.63, 95% CI 1.31, 2.05) times higher than rich youths. The odds of barriers to healthcare access among female youths with no Media exposure were 1.67 (AOR = 1.67, 95%CI 1.41–1.98) times higher than female youths with Media exposure. Female youths who reside in rural areas were 1.63 times more likely to have barriers to healthcare access than urban residents (AOR = 1.63, 95%CI 1.05 to 2.54). Youths from a community with low media exposure were 1.45(AOR = 1.45, 95%CI 1.01–2.08) times more likely to have barriers to health care access compared to those from a community with high media exposure (Table 3).

**Table 3:** Mixed effect analysis of factors associated with barriers to health care access among female youths in Ethiopia, data from 2016 EDHS.

Characteristics		Barriers to healthcare access		Model III (model with individual and community level variables) AOR (95%CI)
		No	Yes	
Age of youths	15–19 year	1090	2291	**0.80 [0.68,0.95]**
	20–24 year	882	1880	**1**
Education status of youths	Uneducated	262	968	**2.26 [1.61,3.18]**
	Primary education	900	2433	**2.21 [1.66,2.95]**
	Secondary	587	597	1.24 [0.93, 1.64]
	Higher education	223	173	**1**
Occupational status of youths	Not working	1101	2527	0.97 [0.84,1.12]
	Working	871	1644	**1**
Wealth index	Poor	376	1650	**1.63 [1.31, 2.05]**
	Middle	299	815	1.21 [0.98, 1.50]
	Rich	1297	1706	1
Marital status	Married	1199	2301	1
	Unmarried	773	1870	**1.43 [1.21,1.70]**
Media exposure	No	640	2399	**1.67 [1.41,1.98]**
	Yes	1331	1773	**1**
Covered by health insurance	No	1834	4009	1.07 [0.78, 1.46]
	Yes	138	162	1
Residency	Urban	787	681	1
	Rural	1185	3490	**1.63 [1.05,2.54]**
Region	Metropolitan	264	192	1
	Large central	1606	3754	1.24 [0.78,1.96]
	Small peripheral	101	226	0.94 [0.55, 1.61]
Community level poverty	Low	761	2585	1
	High	1211	1586	0.79 [0.54, 1.15]
Community-level media exposure	Low	835	2791	**1.45 [1.01,2.08]**
	High	1137	1380	**1**

Significant values are in bold.

### Wealth-related Inequality of barriers to health care access in Ethiopia

**Figure 1:**
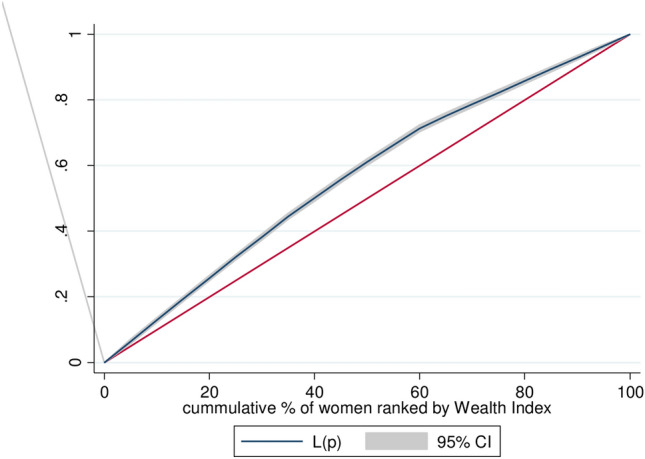
Wealth-related inequality of barriers of health care access among female youths in Ethiopia, EDHS 2016.

In this study, the Errygers normalized concentration index (ECI) and curve were done for EDHS 2016 to assess the wealth-related inequality of barriers to healthcare access among female youths in Ethiopia. The result showed that barriers to health care access were significantly and disproportionately concentrated in the poor households (pro-poor distribution) with [ECI = − 0.282; standard error 0.249, p-value < 0.001]. The graph in Fig. 1 also showed that the distribution line of barriers to healthcare access was above the line of equality. This showed that barriers to healthcare access among female youths in Ethiopia were disproportionately concentrated in poor households (pro-poor distribution).

### Spatial and incremental autocorrelation analysis of barriers to health care access among youth in Ethiopia: based on 2016 EDHS

**Figure 2:**
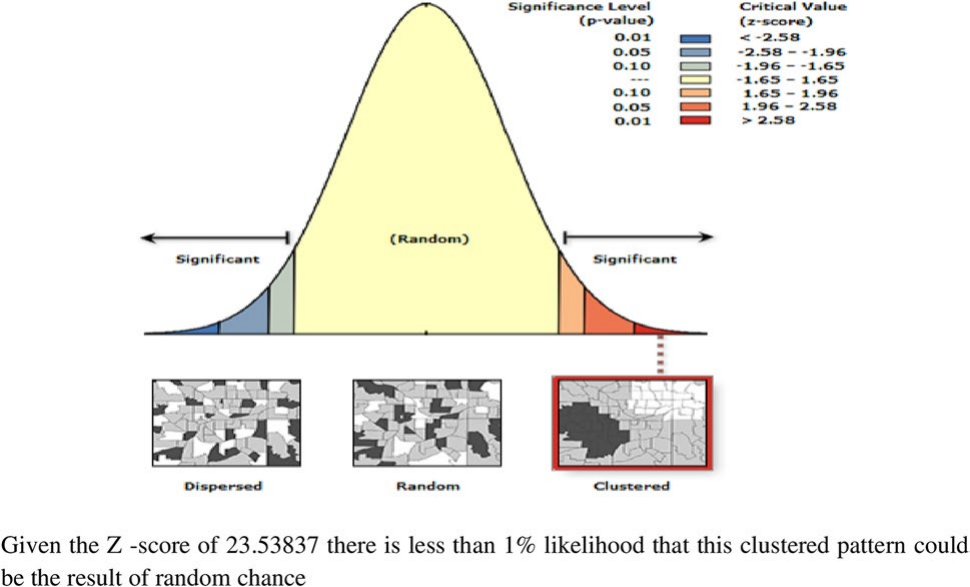
Spatial autocorrelation of barriers to health care access among female youths in Ethiopia, EDHS 2016 plotted using ArcMap 10.7.

Spatial distribution of barriers to health care access among youth in Ethiopia based on 2016. EDHS showed a significant spatial variation across the country over regions, which was found. To be non-random with Global Moran’s I value of 0.72 with (p < 0.0001) (Fig. 2).

The incremental autocorrelation result showed that statistically significant z-scores indicated at one peak distance at 166.821 km; 12.09 (distances; Z-score) for barriers of health care access, where spatial processes promoting clustering were most pronounced by 10 distance bands.

### Spatial distribution of barriers to health care access among youth in Ethiopia: based on 2016 EDHS

**Figure 3:**
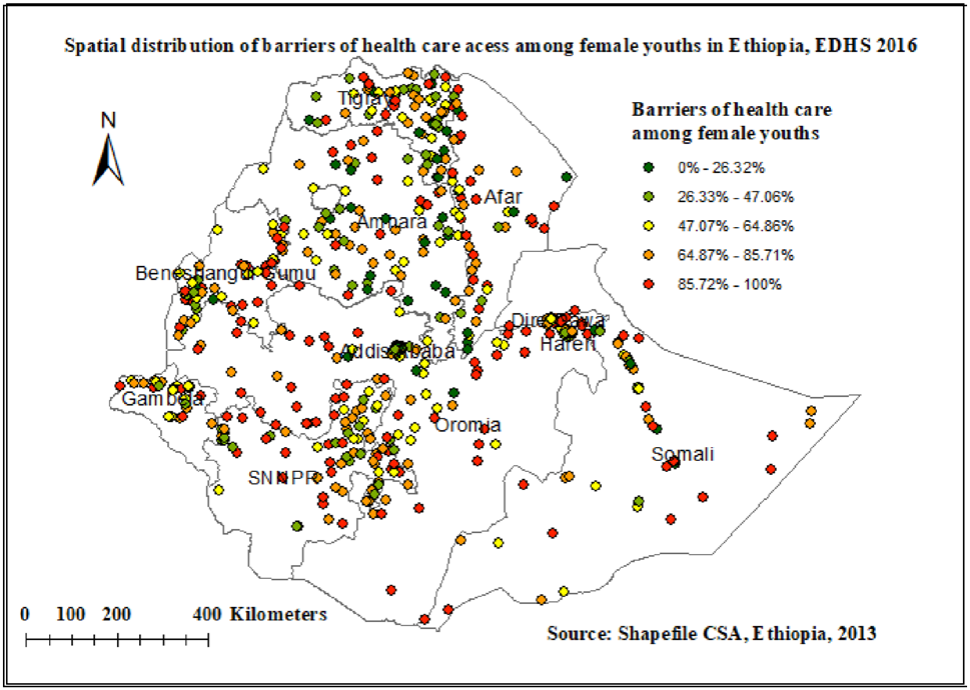
Spatial distribution of barriers to health care access among female youths in Ethiopia EDHS 2016 plotted using ArcMap 10.7.

The spatial distribution of healthcare challenges showed significant spatial variation across the country. As shown in Fig. 3. The red dots indicated the more intense clustering of the proportion of barriers to healthcare access among youths in Ethiopia, whereas the green dots showed a lower proportion of barriers to healthcare access. In this study, the high prevalence of barriers to healthcare access was found in Northern and Western Tigray, Western Afar, the Eastern part of Benishangul Gumuz, Northern Amhara, Northern and Eastern Oromiya Northern and east part of SNNPRs, and the southern part of Somalia regions.

### Kriging interpolation of barriers of health care access among youth in Ethiopia: based on 2016 EDHS

**Figure 4:**
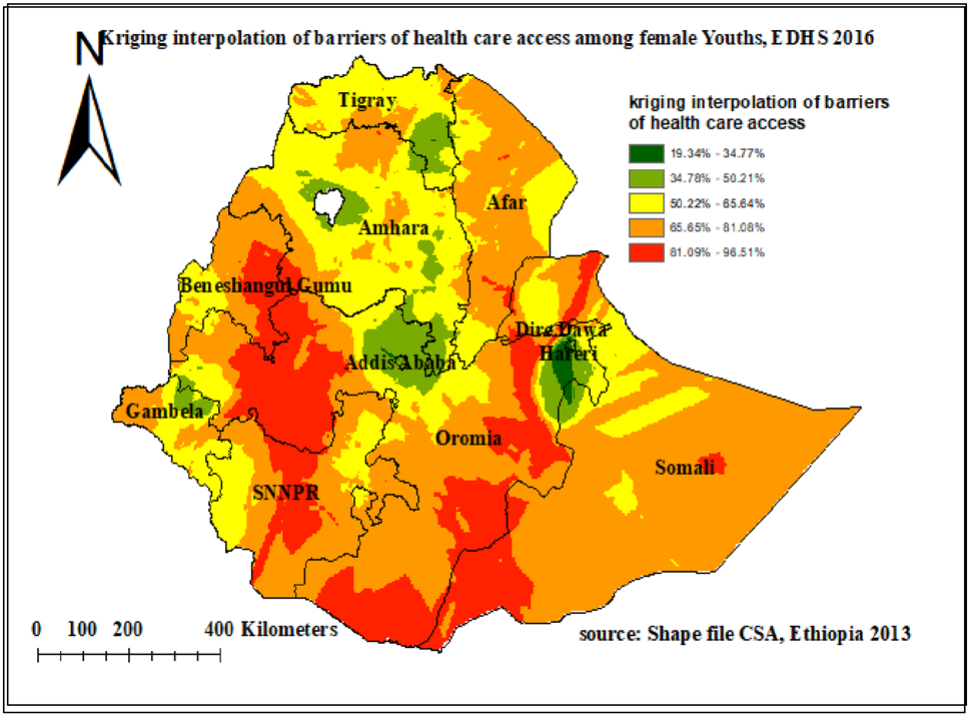
Kriging interpolation of barriers to health care access among female youths in Ethiopia EDHS 2016 plotted using ArcMap 10.7.

Kriging interpolation methods of predicting barriers to health care access among female youth in Ethiopia over the area was increased from green which indicates low- risk to red -colored which indicates high-risk areas. The result revealed that regions such as North Ormiya, East Ormiya and South Ormiya, Dire Dawa, southern Afar, East Benishangul Gumuz, south Benishangul Gumuz, South Somalia and central SNNP regions (South Nation Nationalities and Peoples of Ethiopia) Had predicted higher rates of barriers of health care access (ranging from 81.09 to 96.51%). Whereas the lower predicted barriers to health care access were seen in Addis Ababa, Harari, central and south Amhara, and south Tigray regions (Fig. 4).

### Hot spot analysis (Getis-Ord Gi statistic) of barriers to health care access among youth in Ethiopia

**Figure 5:**
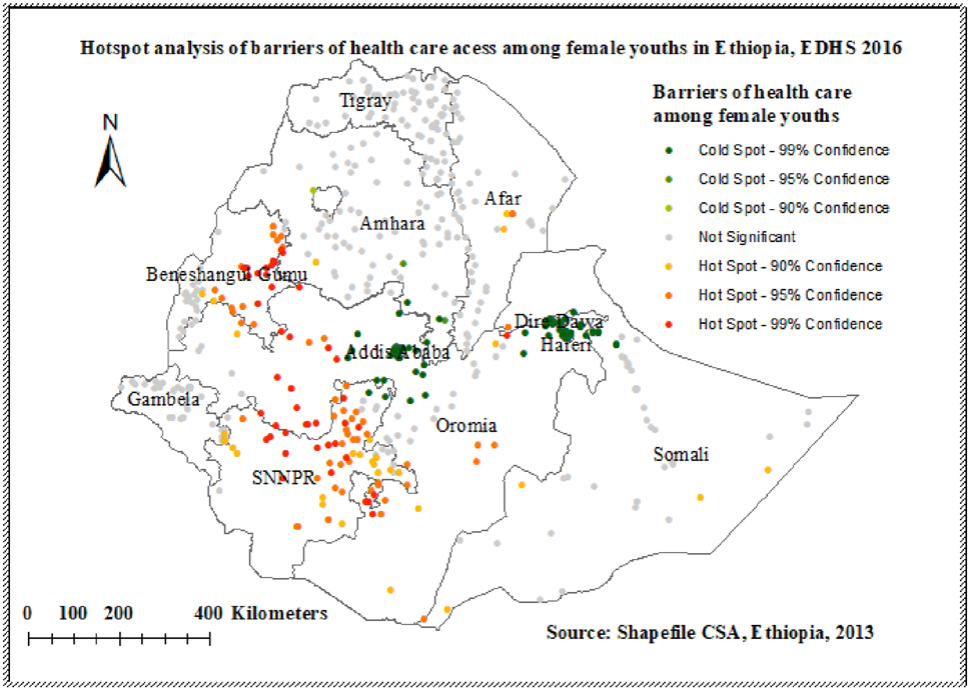
Hot spot area of barriers to health care access among female youths in Ethiopia EDHS 2016 plotted using ArcMap 10.7.

The spatial distribution of barriers to health care access in 2016 EDHS showed that North and south Oromia, Northern, central, and western parts of SNNP (South Nation Nationalities and Peoples of Ethiopia), Western part of Benishangul Gumuz, and central Afar were identified hotspot areas of high barrier of health care access. While cold spot areas were detected in Addis Ababa, Hareri, and Diredewa (Fig. 5).

### Spatial SaTScan statistics analysis of barriers to health care access in Ethiopia

**Figure 6:**
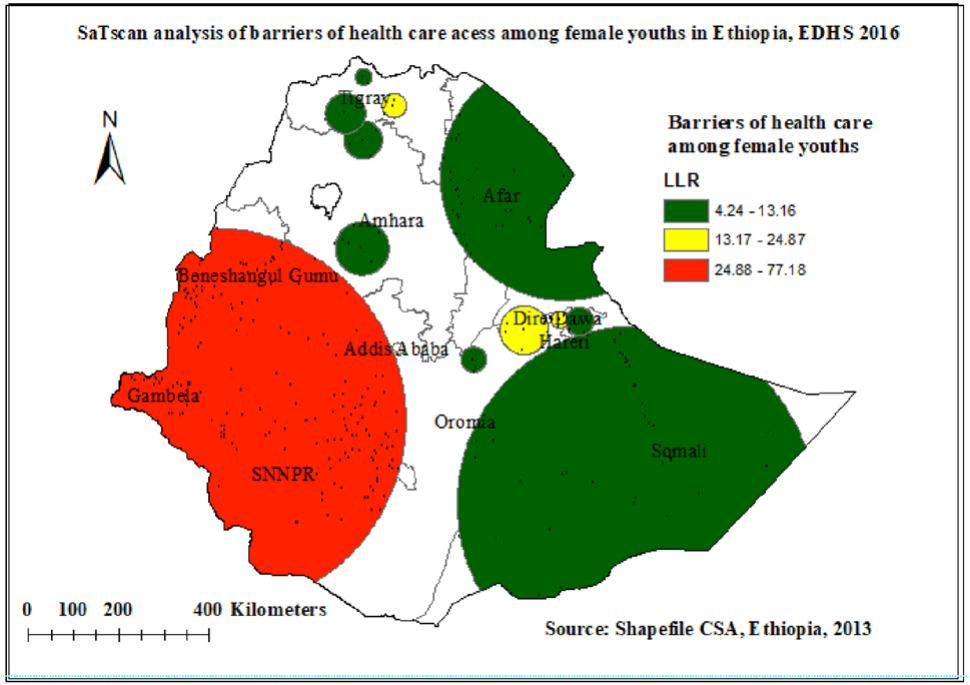
Significant clusters of barriers to health care access spatial window in Ethiopia, EDHS 2016 plotted using Arc Map 10.7.

A spatial scan statistical analysis identified a total of 200 significant primary and secondary clusters. Of these 187 clusters were primary (most likely) clusters which were located in the North Ormiya, Benishangul Gumuz, Gambella, and SNNP regions centered at 7.528248N, 35.063386 E with 426.53 km radius, a Relative Risk (RR) of 1.30, and Log-Likelihood Ratio (LRR) of 77.2, at p-value < 0.0001. It revealed that female youths within the spatial window had a 1.30 times higher risk of experiencing barriers to health care access as compared to female youths outside the spatial window (Table 4, Fig. 6). The secondary clusters were located in Diredawa and central Tigray region. The yellow color circular window (Rings) indicates statistically significant spatial windows containing a high prevalence of barriers to accessing healthcare.

**Table 4:** Significant spatial clusters of barriers of health care access among female youths in Ethiopia, EDHS 2016.

Cluster	Co-ordinate/radius	Population	Cases	RR	LLR	P-value
1	7.528248N,35.063386 E/426.53 km	1866	1349	1.30	77.2	< 0.0001
2	9.339604 N, 41.321415 E/54.20 km	133	117	1.46	24.9	< 0.0001
3	9.339604 N, 41.321415 E/54.20 km	76	72	1.57	24.2	< 0.0001
4	13.848685 N,38.688109 E/27.17 km	41	40	1.61	16.3	< 0.0001
5	5.848373 N, 43.527979 E/394.61 km	356	261	1.22	13.6	< 0.01
6	8.757437 N, 40.299443 E/28.48 km	32	31	1.60	11.9	< 0.01
7	12.401068 N,42.163134 E/274.51 km	320	232	1.21	10.2	0.02

## Discussion

This study was conducted to assess spatial distribution and determinants of barriers to health care access, among female youths in Ethiopia. This study revealed that about 61.3% (95% CI 60.1, 62.5) of Female youths had barriers to healthcare access due to at least one or more of the four reasons, of which difficulty of getting money and distance from health facilities were the most frequently mentioned barriers. The finding of this study is similar to the study done in Benin^14^ and Sub-Sahara Africa^34^. However, barriers to healthcare access among female youths in this study is slightly lower than the study done in South Africa (64.5%)^35^ and Tanzania (65%)^36^. This might be due to socio-cultural among countries which may affect health-seeking behaviors or study population differences. It might be also due to differences in health service availability across study areas.

Moreover, the finding of this study is higher than the Kenya Demographic Health survey (24.21%)^37^. This might be due to the Kenyan government having given more emphasis to the expansion of public facilities and the implementation of an effective healthcare financing policy which increases women’s access to healthcare in the country^37^.

The spatial distribution of barriers to healthcare access among female youths in Ethiopia varied greatly across regions. Significant hotspots of barriers to health care access among female youths were detected in Bensangul, SNNPR, and some parts of the Ormia regions of Ethiopia. The SaTS-can analysis result identified primary clusters found in regions of Bensangul, SNNPR, Northern Oromia, and Gambela region. The possible explanation for regional differences in barriers to healthcare access hot spot areas could be differences in demographic, cultural, and socio-economic factors or it might be due to variations in health care services access across regions of Ethiopia^38^. Furthermore, pastoralists may have limited access to health services due to their high mobility and a strong commitment to cultural values^39^.

Individual and community characteristics attributed to the barriers to health care access among female youths. Thus, Female youths aged 15–19 years were 20% less likely to have barriers to healthcare access than aged 20–24 years. This might be due to youths, 15–19 years old being economically dependent on their family, and health care costs will be covered by family members. Following the age of the youth’s educational status had a significant association with barriers to health care access. Those youths with no formal education and primary education had higher odds of barriers to health care access compared with youths with higher educational status; this finding is supported by studies done in Benin^14^, South Africa^40^, and East Africa^37^. The possible reason for this finding could be better educational status may improve awareness and increase health-seeking behavior^41^ or it might be due to Education is the prominent factor of higher employment opportunities, earning an individual, household, and national economic growth that may in turn increase accessibility for healthcare services^14^.

This study also revealed that the barriers to accessing health care among youths from the poor and middle-wealth classes were higher compared to youths from the lowest-wealth class. The Errygers concentration index and graph also showed that barriers to healthcare access were significantly and disproportionately concentrated in poor households. This finding is in line with studies done in Tanzania^36^, Sub-Saharan Africa^34^, and Ethiopia^41^. The possible reason might be due to a better wealth index may reduce the difficulties of obtaining money to access health care^41^ or accessibility of health services is often influenced by the financial capacity of the households, because both direct costs, like payments for drugs and services, and indirect costs, such as transport cost and unpaid working hours, negatively affect accessibility^14^.

In this study, Marital status also had a significant association with access to healthcare services, unmarried female youths had increased barriers to healthcare access compared with married female youths. This finding is consistent with studies done in Benin^14^, Tanzania^36^, Afar^42^, and Sidama, Ethiopia^43^. The possible explanation might be the financial and psychological support of spouses may improve access to health care^44^.

The residency has a significant association with female youth’s access to health care services. Female youths who reside in rural areas were more likely to have barriers to healthcare access compared with those who were from an urban setting. This finding is consistent with studies done in South Africa^35^, East Africa^37^, and sub-Saharan Africa^34^. This might be due to rural areas being associated with lower geographical accessibility of health facilities^41^, or Rural residency might also impose an extra cost for transportation as well as lack of availability of transportation, and therefore they fail to attain the health facility to utilize health service^37^. Moreover, residing in rural areas have limited access to education and a lower chance of getting health information than women residing in urban areas^34^.

In this study exposure to media was also a significant predictor of barriers to health care access. Female youths with no media exposure had higher barriers to healthcare access than female youths with Media exposure. As well, female youths from a community with low media exposure were more likely to have barriers to healthcare access compared to those female youths from a community with high media exposure. This finding is supported by studies done in South Asia^45^ and Sub-Saharan Africa^34^. This could be due to health information and healthcare being widely disseminated through the mass media, which may improve knowledge, attitudes, and practices related to the use of health services^46^.

The main strength of this study is the use of very huge and representative datasets, which are collected by well-trained data collectors using standard and validated questionnaires that make the findings of this study generalizable for the country. The second strength of this study is the use of multilevel modeling, a model that accounts for the nested/hierarchical nature of the demographic and health survey to get reliable estimates. However, our study is not free from limitations the First limitation, is due to the cross-sectional nature of the data the finding from this study may not build up a genuine causal relationship between the outcome variable and explanatory variables. The information was gathered based on self-report and might have a probability of recall bias. Since we used secondary data quality of the service and health system-related factors were not addressed.

## Conclusion

A significant proportion of female youths faced barriers to healthcare access, of which, money and distance were the commonly perceived barriers. Barriers to healthcare access were distributed non-randomly across Ethiopian regions. Age, educational status, marital status, rural residency low economic status, and media exposure were factors associated with barriers to healthcare access. These findings suggest that program planners and decision-makers should work on improving the country’s economy to a higher economic level to improve the wealth status of the population, promote media exposure, and increase access to education. Policymakers and stakeholders should design targeted intervention programs that take into account youths who reside in areas with highly affected regions of Ethiopia.

## Data Availability

The datasets used and/or analyzed for this study are available from the Demographic and Health Surveys (DHS) Program (https://dhsprogram.com/Data/).

## Abbreviations

ANC: Antenatal care
AOR: Adjusted odds ratio
CI: Confidence intervals
COR: Crude odds ratio
EDHS: Ethiopian Demographic and Health Survey
EA: Enumeration area
ICC: Intra-cluster correlation
OR: Odds ratio
PCV: Proportional change in variance
PNC: Postnatal care
SDG: Sustainable development goal
SRH: Sexual reproductive health
STI: Sexual transmitted infection
WHO: World Health Organization
